# General conditions for spiking neurons and plasticity rules to perform independent component analysis

**DOI:** 10.1186/1471-2202-12-S1-P124

**Published:** 2011-07-18

**Authors:** Carlos SN Brito, Wulfram Gerstner

**Affiliations:** 1 School of Computer and Communications Sciences and Brain-Mind Institute, Ecole Polytechnique Federale de Lausanne (EPFL), Lausanne 1015, Switzerland

## 

Given the many different proposed spiking neuron and plasticity models, it is hard to assess what functional roles their behavior may entail. One possible purpose may be to implement independent component analysis (ICA), which directly relates to sparse coding and finding relevant projections in the input space. Based on the theory of ICA [1], we show how different possible implementations of spiking neurons and spike timing dependent plasticity (STDP) can give similar results. We demonstrate how, given a neuron’s characteristics such as activation rule, STDP model and homeostatic mechanisms, one can assess whether a feedforward two-layer network is able to perform ICA. In particular, we study the behavior of exponential integrate-and-fire neurons with voltage-dependent STDP, a non-linear Hebbian rule [2] that can be related to the BCM theory. Both firing rate and spike-time correlation codes can be used as input, illustrating the flexibility of the plasticity rule in terms of neural coding. When applied to natural image patches, we investigate the capacity of generating Gabor-like receptive fields, as found in the primary visual cortex [3], suggesting a biological implementation of ICA in the brain.
